# Andexanet Alfa in Emergency Cranial Neurosurgical Procedures: A Bicentric Analysis of Efficacy and Safety

**DOI:** 10.1007/s12028-025-02393-z

**Published:** 2025-11-21

**Authors:** Harold F. Hounchonou, Carolin Albrecht, Jonas Jelinek, Florian Wild, Manolis Polemikos, Omar Abu-Fares, Chiara Negwer, Bernhard Meyer, Shadi Al-Afif, Joachim K. Krauss

**Affiliations:** 1 Department of Neurosurgery, Hannover Medical School, Hannover, Germany; 2 Department of Neurosurgery, Klinikum Rechts Der Isar, Technical University Munich School of Medicine, Munich, Germany; 3 Institute of Diagnostic and Interventional Neuroradiology, Hannover Medical School, Hannover, Germany

**Keywords:** Andexanet alpha, Factor Xa inhibition, Intracerebral hemorrhage, Craniotomy, Burr hole, External ventricle drainage

## Abstract

**Background:**

Factor Xa inhibitors are widely used anticoagulants but are associated with a known risk of bleeding complications. In 2018, andexanet alfa was approved as an antidote for the reversal of apixaban and rivaroxaban, demonstrating high efficacy in controlling intracranial hemorrhage. However, data on its use prior to neurosurgical procedures remain limited. Here, we present our experience with andexanet alfa in patients undergoing emergency cranial neurosurgical procedures.

**Methods:**

Here, we present a case series based on a retrospective analysis conducted over a four-year period, identifying patients who underwent cranial neurosurgical procedures while on factor Xa inhibitors and treated with andexanet alfa at two tertiary academic neurosurgical centers in Germany. We reviewed demographic, clinical, and medical data, including age, sex, diagnosis, neurological status, laboratory results, surgical protocols, and imaging studies. The primary end points were (1) the efficacy of andexanet alfa in preventing secondary bleeding during or after surgery (hemostatic efficacy) and (2) its safety concerning ischemic events. Hemostatic efficacy was assessed via postoperative computed tomography scans performed six hours after surgery.

**Results:**

A total of 29 patients (14 female, 15 male) underwent 34 neurosurgical procedures (18 burr-hole craniostomies [BHs] and 16 craniotomies [CRAs]). The patients’ ages ranged from 55 to 94 years (mean 74.82 ± 10.65). The median Glasgow Coma Scale score was 7 at admission. In total, postoperative computed tomography scans revealed no or only minor hematoma in 94% of patients (BH in 94% vs. CRA in 94%; *p* > 0.99). Ischemic events occurred in 9 patients, including 8 cases of cerebral ischemia and one case of mesenteric ischemia (BH: 28%; CRA: 31%; *p* > 0.99). No cases of deep vein thrombosis or pulmonary embolism were recorded. The use of additional hemostatic agents was associated with the occurrence of ischemic events (*p* = 0.04, 95% confidence interval 1.11–122.77, odds ratio 11.7). Overall, in-hospital mortality was 28% in total.

**Conclusions:**

Our findings suggest that andexanet alfa is highly effective in preventing secondary bleeding during neurosurgery in patients on factor Xa inhibitors. However, the risk for ischemic events must be carefully considered.

## Introduction

Factor Xa inhibitors are widely used anticoagulants for the prevention of ischemic events in patients with atrial fibrillation (AF) and for the treatment of venous thromboembolism [1, 2]. Their use, however, is associated with a notable risk of bleeding, including intracerebral hemorrhage (ICH) [3]. ICH occurs in approximately 0.3–0.5% of patients on factor Xa inhibitors annually [4, 5]. To reverse the anticoagulant effect of factor Xa inhibitors, the antidote andexanet alfa was developed and showed promising results in two randomized, double-anonymized, placebo-controlled phase III trials (ANNEXA-A and ANNEXA-R), which led to its approval by the US Food and Drug Administration in May 2018 [6, 7]. Additionally, andexanet alfa has been shown to be highly effective in the conservative management of ICHs in a phase IIIb/IV trial (ANNEXA-4), which included 331 patients [8]. In the more recent study (ANNEXA- I) evaluating the efficacy of andexanet alfa compared with standard conservative management in ICH, andexanet alfa demonstrated superior control of hematoma expansion [9]. One of its downsides, however, is that its use has been associated with an increased risk of ischemic events [8, 9].

In addition to conservative measures, emergency neurosurgical interventions can be required as life-saving procedures or to improve functional outcome in patients with ICH [10]. Despite the growing body of evidence on andexanet alfa’s efficacy in reversing factor Xa inhibition, data on its use in surgical settings remain limited [11–14].

Here, we present a series of patients who underwent emergency trepanation following the reversal of factor Xa inhibitor activity with andexanet alfa. We specifically evaluate its hemostatic efficacy in preventing secondary intraoperative or postoperative bleeding and assess its safety regarding the occurrence of ischemic events.

## Methods

### Study Design

This study presents a case series based on a retrospective analysis conducted between 2020 and 2024 at two tertiary academic neurosurgical centers in Germany: Hannover Medical School and the Technical University of Munich School of Medicine. Patients who underwent emergency surgery for intracranial hemorrhage in whom factor Xa inhibitors were administered within 24 h prior to surgery were identified from our databases. Demographic, clinical, and imaging data were collected for each patient. The clinical data included the underlying pathology (diagnosis at admission; for example, subdural hematoma [SDH] or ICH) and indication for emergency surgery, the rationale for anticoagulation therapy, the specific factor Xa inhibitor used, concomitant use of antiplatelet drugs, comorbidities, Glasgow Coma Scale (GCS) at admission, dose of andexanet alfa (high or low), use of blood products, type of surgical procedures performed, surgery duration, hemoglobin levels at admission and on the first postoperative day, and neurological status at discharge and at three-month follow-up (assessed using the modified Rankin Scale [mRS] ranging from 0 to 6; 0: no symptoms, 1: no significant disability, 2: slight disability, 3: moderate disability, 4: moderately severe disability, 5: severe disability, 6: dead) [15]. In addition, operative reports were reviewed for documentation of excessive intraoperative blood loss or difficulty achieving hemostasis. Imaging data included preoperative and postoperative computed tomography (CT) scans. Furthermore, preoperative and postoperative hematoma volumes were assessed using semiautomated three-dimensional region-of-interest–based volumetry.

The primary end points of the study were (1) the hemostatic efficacy of andexanet alfa in preventing secondary bleeding during or after surgery (within six hours postoperatively) and (2) the safety profile, particularly with regard to ischemic events, including cerebral ischemia, clinically significant deep vein thrombosis, and pulmonary embolism. The efficacy was determined based on the presence of hematoma at the surgical site on the postoperative CT scan (six hours after surgery), defined as any new hyperdense blood collection along the surgical trajectory—including epidural, subdural, or parenchymal components. When present, hematomas were classified as either minor or major based on radiological criteria. Minor hematoma was defined as a hyperdense collection measuring < 10 mm in maximal diameter without associated mass effect. Major hematoma was defined as a hyperdense collection ≥ 10 mm in diameter or any hematoma exhibiting mass effect on adjacent structures. The use of hematoma diameter, rather than volumetric assessment, was chosen to reflect routine clinical practice in neurosurgery, in which volumetry is not consistently available. Given that postoperative bleeding in our cohort could occur in various compartments—epidural, subdural, or parenchymal—we applied the most conservative threshold for clinical relevance, namely a diameter of ≥ 10 mm, which is widely accepted as an indication for surgical evacuation in SDHs [16]. To identify ischemic events, the medical records of all patients were systematically reviewed by members of the study team (H.F.H., J.J., and C.A.). In addition, all imaging studies were independently assessed by a board-certified neuroradiologist (O.A.-F.), and findings were cross-referenced to ensure consistency. The same review process was applied uniformly to all patients included in the study. Furthermore, intraoperative blood loss was indirectly evaluated by analyzing preoperative and postoperative hemoglobin levels.

### Data Analysis

In addition to descriptive statistics, data were compared between patients undergoing burr-hole craniostomy (BH) and those undergoing craniotomy (CRA) to assess whether the hemostatic efficacy of andexanet alfa varied depending on the invasiveness, the extent, and the duration of the surgical procedure. Continuous variables (age, hemoglobin levels, and surgery duration) are expressed as mean ± standard deviation. GCS and mRS are expressed as median (and range). Categorical variables, including sex, andexanet alfa dose (low vs. high), efficacy (no or minor hematoma vs. major hematoma), and the presence of arterial hypertension, diabetes mellitus, coronary artery disease, and previous ischemic events, were analyzed using Fisher’s exact test. Age was compared using Student’s *t*-test, and hemoglobin levels were analyzed by repeated measures analysis of variance (ANOVA). Preoperative and postoperative hematoma volumes are presented as mean ± standard deviation and compared using a paired *t*-test. Multivariable analyses were performed with an online statistical tool (https://statisty.app/logistic-regression-calculator, accessed on March 19, 2025); variables included in the models were selected based on their known or potential clinical relevance to outcomes. All other statistical analyses were performed using GraphPad Prism (GraphPad Prism 10.4.1 [532], Macintosh version, by Software MacKiev, 1994–2024, GraphPad Software, LLC). Statistical significance was defined as *p* < 0.05.

## Results

### Cohort Description

A total of 33 patients received andexanet alfa prior to surgical intervention at our institutions. Of these, four patients who underwent spinal procedures were excluded. The final study cohort comprised 29 consecutive patients undergoing 34 neurosurgical cranial procedures following andexanet alfa administration. The cohort comprised 15 men and 14 women, with a balanced distribution across groups (BH: 9 men, 9 women; CRA: 8 men, 8 women; *p* > 0.99). Patients’ ages ranged from 55 to 94 years (mean: 74.92 ± 10.65 years), with no significant difference between groups (BH: 73.94 ± 11.41 years; CRA: 74.69 ± 10.72 years; *p* = 0.85). Regarding comorbidities, arterial hypertension was present in 55% of patients, diabetes mellitus was present in 14%, and coronary artery disease was present in 14%, with no significant differences between groups (arterial hypertension: *p* > 0.99; diabetes mellitus: *p* = 0.65; coronary artery disease: *p* = 0.65). A history of ischemic events was reported in ten patients, with a similar distribution between groups (BH: 33%; CRA: 25%; *P* = 0.72). An overview of patients’ data and group comparisons is provided in Table 1.

**Table 1:** Data summary and group comparison

	All (N = 34)	BH (N = 18)	CRA (N = 16)	p-value (CRA vs. BH)
Age (years)	74.92 ± 10.65	73.94 ± 11.41	74.69 ± 10.72	0.85
Sex (m/w)	15/14	9/9	8/8	> 0.99
GCS at admission (min–max)	7 (3–15)	7 (3–14)	8 (3–15)	0.42
Postoperative CT-findings (no or minor hematoma /major hematoma)	32/2	17/1	15/1	> 0.99
Ischemic events (yes/no)	10/34	5/13	5/11	> 0.99
mRS at discharge (min–max)	5 (1–6)	5 (3–6)	5 (1–6)	0.13
*Comorbidities*				
Arterial hypertension (yes/no)	19/15	10/8	9/7	> 0.99
Diabetes mellitus (yes/no)	5/29	2/16	3/13	0.65
Coronary artery disease (yes/no)	5/29	2/16	3/13	0.65
Ischemic events (yes/no)	10/24	6/12	4/12	0.71

At presentation, 64% of patients were in coma or sopor. The median GCS was 7 (BH: 7; CRA: 8; *p* = 0.42). Emergency surgery was indicated for ICH (*n* = 13), SDH (*n* = 9), severe traumatic brain injury (*n* = 3), and aneurysmal subarachnoid hemorrhage (aSAH) (*n* = 3); one patient had both SDH and aSAH. A total of 16 CRAs, including 3 decompressive hemicraniectomies, were performed. Furthermore, 18 BHs were performed, including procedures for external ventricular drain (EVD) placement in 12 patients and intracranial pressure (ICP) monitor insertion in 3 patients. EVDs were placed to manage hydrocephalus secondary to intraventricular hemorrhage (*n* = 9) or aSAH (*n* = 3), whereas ICP monitors were inserted in patients with severe traumatic brain injury for continuous ICP monitoring. A total of five patients underwent two neurosurgical procedures. In three cases, an initial BH was performed for EVD placement, followed by a CRA for hematoma evacuation. In the remaining two cases, EVD revision was required because of initial catheter misplacement. Notably, no revision surgeries were performed for postoperative bleeding. The surgery duration ranged from 10 to 231 min. The interval from andexanet alpha bolus to skin incision for surgery was estimated based on the time from initial CT scan to skin incision and ranged from 1 to 14 h (median 1 h).

The most commonly used factor Xa inhibitor was apixaban, prescribed to 21 patients (74%), followed by rivaroxaban in 7 patients (24%) and edoxaban in 1 patient. The indication for anticoagulation was AF in 25 patients (86%), pulmonary artery embolism in 3 patients (10%), and a transient ischemic attack in 1 patient. According to the manufacturer’s guidelines (Portola Pharmaceuticals, South San Francisco, CA), Andexanet alfa was administered at a low dose (intravenous bolus of 400 mg followed by an infusion of 480 mg over 2 h) to 21 patients and at a high dose (intravenous bolus of 800 mg followed by an infusion of 960 mg over 2 h) to 8 patients (BH: 67% low dose; CRA: 69% low dose; *p* > 0.99). A concomitant use of antiplatelet drugs was reported in 2 patients (acetylsalicylic acid: *n* = 1; clopidogrel: *n* = 1). Additional hemostatic agents, including tranexamic acid, prothrombin complex concentrates (PCCs), thrombocyte concentrates (TCs), fibrinogen concentrates, and fresh frozen plasma, were administered in seven cases. Specifically, as detailed in Table 2, patient 2 was administered tranexamic acid preoperatively as a prophylactic measure. Patient 10 and patient 11 both received PCC preoperatively because of a low Quick value in the former and as a prophylactic measure in the latter. Patient 14 was administered TC on postoperative day 1 for thrombocytopenia. Patient 18 received both fresh frozen plasma and PCC preoperatively because of a low Quick value, followed by TC, PCC, and fibrinogen concentrate four hours postoperatively in response to thrombocytopenia, low Quick value, and fibrinogen deficiency, respectively. Patient 19 received PCC on the first postoperative day for a reduced Quick value. Finally, patient 29 received PCC preoperatively as a prophylactic measure. No patient received a second dose of andexanet alfa. All patients were on low-molecular-weight heparins for thrombosis prevention starting at the first or second postoperative day. Baseline characteristics and outcomes are detailed in Table 2.

**Table 2:** Demographic data, baseline characteristics and outcomes

ID	Age	Sex	Type of craniocerebral hemorrhage	GCS at admission	Indication for anticoagulation	FXa inhibithor	AA dose	Additional hemostatic agent	Time tom surgery (hours)	Surgery	Postoperative CT findings	Ischemic event	mRS at discharge
1	91	W	cSDH (30 mm)	8	AF	APX	Low	–	1	BH + evacuation	Minor hematoma	–	3
2	81	W	Severe TBI, brain edema, CHC	7	AF	APX	Low	TXA	11	BH + ICP monitor placement	No hematoma	Cerebral ischemia on day 1	6 (death on day 3)
3	91	M	Severe TBI, brain edema	3	AF	APX	Low	–	3	BH + ICP monitor placement	No hematoma	–	6 (death on day 14)
4	82	W	Severe TBI, CHC	3	AF	EDX	Low	–	3	BH + ICP monitor placement	No hematoma	–	5
5	83	M	ICH (thalamus, 30 mm) + IVH ICH score: 3	14	AF	APX	High	–	6	BH + EVD placement	Minor hematoma	Cerebral ischemia on day 5	4
6	65	W	aSAH (Fisher grade 4)	7	PAE	RVX	Low	–	1	BH + EVD placement	Minor hematoma	Cerebral ischemia on day 4	4
7	75	W	ICH (basal ganglia, 34 mm) + IVH ICH score: 3	9	AF	APX	Low	–	1	BH + EVD placement	No hematoma	–	6 (death on day 7)
8	77	M	ICH (basal ganglia, 28 mm) + IVH ICH score: 1	9	AF	APX	Low	–	4	BH + EVD	Minor hematoma	–	6 (death on day 18)
9	77	W	aSAH, Fisher grade 4	3	AF	APX	High	–	1	BH + EVD	Major hematoma	–	6 (death on day 10)
10	75	M	ICH (frontal, 30 mm) ICH score: 2	7	AF	APX	Low	PCC	1	BH + EVD	Minor hematoma	–	5
11	57	W	ICH (cerebellar, 30 mm) ICH score: 2	7	PAE	APX	Low	PCC	1	1) BH + EVD 2) CRA + evacuation	1) No hematoma 2) No hematoma	Cerebral ischemia on day 4	3
12	94	M	Bilateral cSDH (left: 25 mm, right: 21 mm)	13	AF	APX	Low	–	6	BH + evacuation	Minor hematoma	–	6 (death on day 2)
13	64	W	ICH (basal ganglia, 39 mm) + IVH ICH score: 2	7	AF	RVX	Low	–	2	1) BH + EVD 2) Revision due to misplacement	No hematoma	Acute mesenteric ischemia on day 1	6 (death on day 8)
14	61	M	ICH (basal ganglia, 33 mm) ICH score: 1	13	AF	APX	High	TC	1	1) BH + EVD 2) Revision due to misplacement	1) No hematoma 2) No hematoma	–	4
15	67	M	ICH (cerebellar, 48 mm) ICH score: 4	7	AF	APX	High	–	2	1) BH + EVD 2) CRAy + evacuation	1) Minor hematoma 2) Minor hematoma	–	5
16	66	M	ICH (cerebellar, 55 mm) + IVH ICH score: 3	3	AF	RVX	High		1	1) BH + EVD 2) CRA + evacuation	1) No hematoma 2) No hematoma	–	5
17	55	M	aSDH (22 mm)	13	AF	APX	Low	–	2	Decompressive hemicraniectomy + evacuation	Minor hematoma	–	4
18	81	M	aSDH (11 mm)	3	PAE	RVX	Low	PCC, TC, FC, FFP	1	Decompressive hemicraniectomy + evacuation	Major hematoma	–	5
19	76	M	aSAH (Fisher grade 4) + aSDH (16 mm)	3	AF	APX	Low	PCC	1	Decompressive hemicraniectomy + clipping + evacuation	Minor hematoma	Cerebral ischemia on day 5	6 (death on day 35)
20	80	W	aSAH (Fisher grade 4)	7	AF	APX	High	–	1	CRA + Clipping + EVD placement	No hematoma	Cerebral ischemia on day 1	5
21	76	W	ICH (cerebellar, 41 mm) ICH score: 2	14	AF	APX	Low	–	1	CRA + evacuation	Minor hematoma	–	4
22	87	W	ICH (temporal, 50 mm) ICH score: 2	13	AF	APX	Low	–	13	CRA + evacuation	No hematoma	–	4
23	84	W	aSDH (24 mm)	6	AF	RVX	High	–	1	CRA + evacuation	No hematoma	–	5
24	72	W	aSDH (12 mm)	15	AF	RVX	Low	–	2	CRA + evacuation	No hematoma	–	2
25	70	M	aSDH (18 mm)	14	AF	APX	Low	–	4	CRA + evacuation	No hematoma	–	1
26	70	W	ICH (temporal, 52 mm) ICH score: 2	14	AF	APX	Low	–	4	CRA + evacuation	Minor hematoma	–	3
27	87	M	ICH (parietal, 37 mm) ICH score: 2	14	AF	APX	Low	–	14	CRA + evacuation	No hematoma	–	4
28	73	W	aSDH (19 mm)	3	AF	RVX	Low	–	1	CRA + evacuation	No hematoma	Cerebral ischemia on day 1	6 (death on day 13)
29	94	M	aSDH (15 mm)	9	TIA	APX	High	PCC	2	CRA + evacuation	Minor hematoma	Cerebral ischemia on day 6	6 (death on 15)

aSAH: aneurysmal subarachnoid hemorrhage, aSDH: acute subdural hematoma, APX: Apixaban, BH: burr-hole craniostomy, CHC: cerebral hemorrhagic contusion, CRA: craniotomy, cSDH: chronic subdural hematoma, EDX: Edoxaban, EVD: external ventricle drainage, FC: fibrinogene concentrate, FFP: fresh frozen plasma, ICH: intracerebral hematoma, IVH: intraventricular hematoma PAE: pulmonary artery embolism, PCC: prothrombin complex concentrate, RVX: Rivaroxaban, TBI: traumatic brain injury, TC: thrombocyte concentrate, TIA: transient ischemic attack, TXA: tranexamic acid

### Hemostatic Efficacy and Intraoperative Blood Loss

**Fig. 1:**
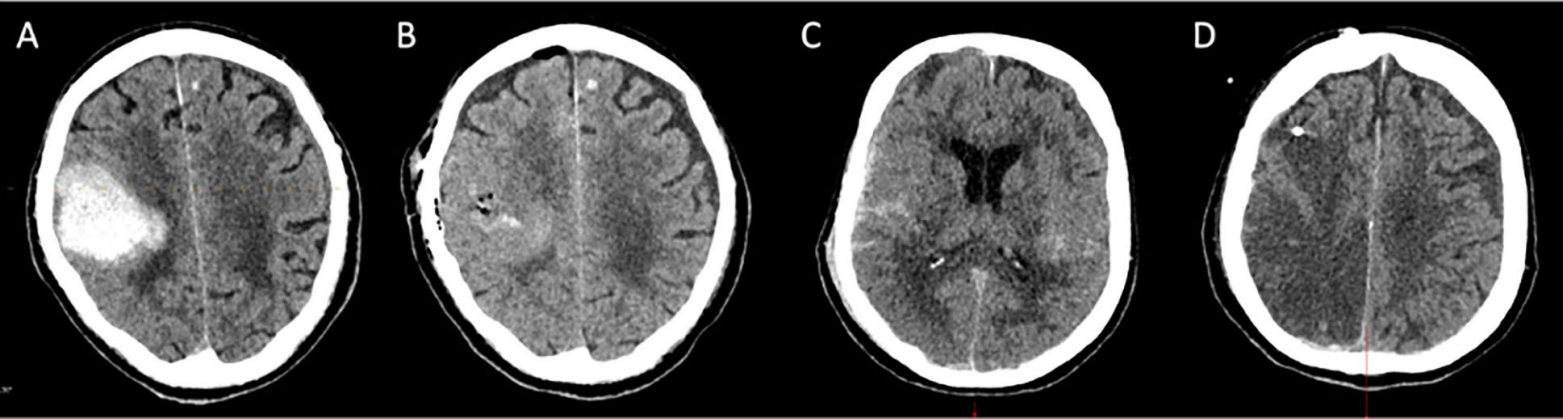
Representative preoperative and postoperative CT scans of two patients. (**A**) Preoperative CT scan of patient 27, showing a 37 mm parietal intracranial hemorrhage. (**B**) Postoperative CT scan of the same patient, demonstrating successful hematoma evacuation with no significant residual hemorrhage at the surgical site. (**C**) Initial CT scan of patient 2 with severe traumatic brain injury. (**D**) Follow- up CT scan one day after intracranial pressure monitor placement, showing no procedure-related bleeding but revealing an ischemic stroke in the posterior and middle cerebral artery territories. CT, computed tomography

Postoperative CT scans showed no hematoma in 20 procedures (59%) (Fig. 1B), minor hematoma in 12 procedures (35%), and major hematoma in 2 procedures (6%). There was no significant difference between the groups (BH: no or minor hematoma in 94%; CRA: no or minor hematoma in 94%; *p* > 0.99). Also, hemostatic efficacy was comparable between patients receiving apixaban and those receiving rivaroxaban, with no or minor hematoma observed in 95% and 86% of cases, respectively (*p* = 0.44). Additionally, a comparison between patients who received a high dose versus a low dose of andexanet alfa revealed no significant difference in hemostatic efficacy (high dose: no or minor hematoma in 88%; low dose: no or minor hematoma in 95*%; p* = 0.48).

**Fig. 2:**
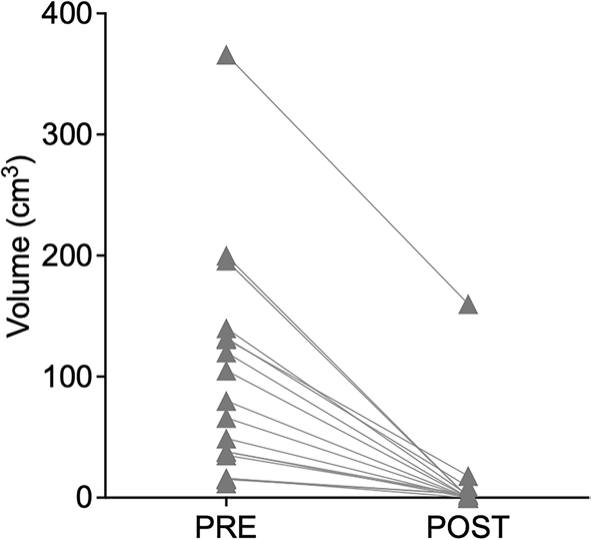
PRE and POST hematoma volume in 17 patients who underwent hematoma evacuation. POST, postoperative, PRE, preoperative

Preoperative hemoglobin levels were 12.86 ± 1.93 mg/dL, and postoperative levels were 11.07 ± 2.01 mg/dL. Repeated measures ANOVA showed a significant drop in hemoglobin levels after both BHs and CRAs (ANOVA time factor: *p* < 0.0001), with no significant difference between the two procedures (ANOVA interaction factor: *p* = 0.68). In patients who underwent surgery for hematoma evacuation (SDH: *N* = 10; ICH: *N* = 7), the mean preoperative volume was 110.64 ± 88.19 cm^3^ (SDH: 149 ± 93 cm^3^; ICH: 37 ± 18 cm^3^), which decreased postoperatively to 13.14 ± 38.94 cm^3^ (*p* < 0.0001, see Fig. 2). No red blood cell transfusions were required intraoperatively or during the first 48 h postoperatively. Review of the operative reports did not reveal any documentation of abnormal intraoperative bleeding or unexpected blood loss.

### Safety and Functional Outcome

Ischemic events occurred in nine patients after a total of ten surgical procedures (Fig. 1D). These included eight cases of cerebral ischemia and one case of acute mesenteric ischemia. The time from surgery to detection of ischemic events ranged from one to six days (median of three days). The distribution between groups was similar (BH: 28%; CRA: 31%; *p* > 0.99). A subgroup comparison between patients receiving apixaban and those receiving rivaroxaban did not reveal a significant difference in the occurrence of ischemic events (apixaban 29% vs. rivaroxaban 43%; *p* = 0.65). Similarly, no significant difference was observed when comparing patients who received a high dose versus a low dose of andexanet alfa (high dose 37% vs. low dose 29%; *p* = 0.68).

A total of six patients with ischemic events (67%) had AF. Cerebral ischemia was detected following asystole and resuscitation in two patients and in the context of aSAH in three other patients. Among the three patients with aSAH, one patient developed vasospasm during the clinical course; however, cerebral infarction was already evident on the first postoperative day. In the remaining two patients, infarctions were detected on postoperative days 4 and 5, respectively, without evidence of vasospasm in the course. Four patients exhibited multifocal cerebral ischemia. No cases of clinically significant pulmonary artery embolism, myocardial infarction, or deep vein thrombosis were found. Among the 7 patients who received additional hemostatic agents, 4 developed ischemic events (57%), compared with 5 out of 22 patients (23%) who did not receive additional agents. Multivariable analysis, including age, sex, GCS, and the use of additional hemostatic agents, showed that the use of additional hemostatic agents was independently associated with the occurrence of ischemic events (*p* = 0.04, 95% confidence interval 1.11–122.77, odds ratio 11.7).

At discharge, the median mRS was 5 (ranging from 1 to 6). In-hospital mortality was 28%, with most deaths occurring after extubation related to prolonged coma and lack of neurological improvement. The patient with acute mesenteric ischemia died because of subsequent sepsis. Multivariable analysis, including age, sex, GCS, and the occurrence of ischemic events, showed only age as an associated risk factor for in-hospital death (*p* = 0.04, 95% confidence interval 1–1.21, odds ratio 1.1). The occurrence of ischemic events was not significantly associated with in-hospital death (*p* = 0.09). Time from surgery to death ranged from 2 to 35 days (median 11.5 days). Out of 19 discharged patients, 4 were lost to follow-up, and a 3-month mRS was available in 15. Among these, the median 3-month mRS was 4 (ranging from 0 to 6).

## Discussion

This study represents the largest series to date of patients undergoing cranial neurosurgical procedures after receiving andexanet alpha for reversal of factor Xa inhibitors. In 94% of patients, no or only minor hematoma was detected on postoperative CT scans. However, ischemic events occurred in 31% of patients during the clinical course. In-hospital mortality was 28%, and the median mRS at 3-month follow-up was 4. Outcomes were comparable between CRA and BH. Furthermore, group comparison of apixaban versus rivaroxaban and high versus low dose revealed no significant difference in hemostatic efficacy or occurrence of ischemic events.

The majority of published real-world experiences regarding the use of andexanet alfa have focused on its efficacy in controlling major bleeding in nonoperative cases, as patients requiring surgery were excluded from the ANNEXA-4 trial [8]. Since its US Food and Drug Administration approval in 2018, several investigators have sought to validate the findings of ANNEXA-4 in clinical practice [17–20]. For instance, Sarhan et al. conducted a meta-analysis of 16 studies evaluating the efficacy of andexanet alfa for conservative management of factor Xa inhibitor–associated ICH in 598 patients, reporting successful anticoagulant reversal in 80.2% of cases [21]. The approval of andexanet alpha, however, did not include its use in the preoperative setting.

Neurosurgeons may encounter emergencies in patients on factor Xa inhibitors who require surgery, such as evacuation of ICH or EVD placement. Thus far, there is no uniform consensus on the clinical management of these patients. Prior to the approval of andexanet alfa, several investigators reported the use of four-factor PCC for the reversal of factor Xa inhibitors before invasive procedures; however, the available data remain limited [22, 23]. Similarly, only a few studies have reported their experience with the preoperative use of andexanet alfa [11–14]. The largest of these, by Bradshaw et al., included 44 patients undergoing various intracranial and extracranial surgical procedures, of whom 15 had neurosurgical interventions (6 CRAs and 9 EVD placements) [11]. They reported excellent or good hemostasis (hematoma expansion after surgery ≤ 35% of baseline) in 80% of patients [11]. Similarly, Ammar et al. published a series of 12 patients with factor Xa–related ICH undergoing EVD placement under andexanet alfa, with no tract hemorrhages observed [12]. In a series of 39 patients with factor Xa inhibitor–associated intracranial hemorrhage, Giovino et al. reported that 5 patients underwent neurosurgical procedures (4 CRAs and 1 EVD placement) following andexanet alfa reversal, and there was no evidence of rebleeding or hematoma expansion after surgery [13].

Here, we present the largest series to date of patients undergoing neurosurgical emergency procedures with andexanet alfa, including 29 patients and 34 surgical procedures (16 CRAs including 3 decompressive hemicraniectomies and 18 burr-hole CRAs, including insertion of EVD or ICP monitor in 15 cases). In the majority of cases, postoperative CT scans revealed no or only minor hematoma both in CRAs and BHs (94%). Furthermore, intraoperative blood loss was not higher in CRAs compared with BHs. Surgeons did not report any abnormal intraoperative bleeding, suggesting that the observed hemoglobin drop may be attributable to factors such as parenteral hydration or central venous access [24]. These findings corroborate those of the above-mentioned studies and suggest that the hemostatic efficacy of andexanet alfa in preventing intraoperative or postoperative bleeding is comparable to its efficacy in the conservative management of intracranial hematoma, as demonstrated in ANNEXA-4 and ANNEXA-I, in which good or excellent hemostatic efficacy—defined as ≤ 35% hematoma expansion within 12 h—was achieved in 76–82% of cases [8, 9].

A key concern regarding the use of andexanet alfa is the associated risk of ischemic events. The exact mechanisms of ischemic events in patients receiving andexanet alpha are not fully understood. It has been hypothesized that patients might have an increased risk of thrombotic events because of rapid reversal of anticoagulation or that andexanet alpha might have a direct procoagulant effect [9]. In the ANNEXA-4 and ANNEXA-I trials, ischemic events were reported in 10% of patients [8, 9]. A meta-analysis of 525 patients with conservatively managed intracranial hemorrhages found ischemic events in 14% of cases [25]. In our cohort, the incidence of ischemic events was 31%, which is notably higher than in ANNEXA-4 and ANNEXA-I but comparable to findings from Bradshaw et al., who also included patients undergoing surgical procedures and reported ischemic events in 27.3% of cases [11]. This difference may be related to additional postoperative hypercoagulability as previously described in surgical patients [26, 27]. Furthermore, our analysis revealed that the use of additional hemostatic agents was associated with ischemic events. In another publication of Bradshaw and colleagues, a series of five patients was reported, who had received four-factor PCC and andexanet alpha for reversal of factor Xa inhibitors with ischemic events in two out of five cases [28]. In our cohort, additional hemostatic agents were administered in four cases based on laboratory findings, whereas in three other cases, they were given as a prophylactic safety measure because of the lack of established data on andexanet alfa use in the perioperative setting. This reflects the current clinical uncertainty surrounding optimal management in this setting and underscores the importance of studies to provide more data and guide future standardized treatment strategies. It is important to note that there were potential confounders in our cohort, such as SAH and asystole, both of which have been described to be independently associated with cerebral ischemia [29, 30].

Regarding mortality, our cohort exhibited an in-hospital mortality rate of 28%, whereas the ANNEXA-4 study reported a 30-day mortality rate of 15% [8]. Other studies have reported mortality rates of 24% in conservatively managed patients and 34.1% in surgically managed patients [11, 25]. Because indications for surgery include symptomatic lesions, marked mass effect, and neurologic deterioration [31], it has to be considered that such patients are in a worse neurological condition preoperatively, which explains higher mortality. Furthermore, our data show that mortality is associated with higher age.

This study has several limitations that warrant consideration. First, the cohort size was relatively small and heterogeneous, encompassing patients with varying intracranial pathologies and surgical interventions, which limits the generalizability of the findings. Second, the exact timing of the last administered dose of factor Xa inhibitors was not consistently documented, which would have been important to assess the residual anticoagulant activity at the time of surgery. Third, the absence of a control group—such as patients receiving alternative reversal strategies or no reversal—restricts direct comparison and limits definitive conclusions regarding the efficacy of andexanet alfa in this setting. Additionally, hemostatic efficacy was assessed via CT imaging at six hours postoperatively, and minor hematomas detected on these scans could partially represent residual preoperative hematoma rather than new bleeding. Although the inclusion of two tertiary academic centers strengthens the generalizability of our findings, the bicentric nature of the study also introduces potential sources of bias. Differences in institutional protocols, clinical decision-making, documentation practices, and resource availability may have influenced patient selection, perioperative management, and reporting of outcomes. Although data collection followed a standardized approach across both sites, unmeasured intercenter variability cannot be fully excluded and may contribute to heterogeneity in the data set. Although it would have been of interest to perform a univariable subgroup analysis to explore the association between the use of additional hemostatic agents and ischemic events, the small size of this subgroup (*n* = 7) limited the statistical validity of such an approach. However, multivariable analysis revealed an association between additional hemostatic therapy and ischemic events, suggesting the need for further study in larger patient cohorts. Finally, no systematic screening for asymptomatic deep vein thrombosis was performed.

## Conclusions

Our data suggest favorable hemostatic efficacy of andexanet alpha in preventing secondary bleeding in patients undergoing CRA or BH (with and without EVD or ICP monitor placement) after anticoagulation with factor Xa inhibitors. There is a notable risk, however, of ischemic events that should be considered, especially when additional hemostatic agents are used. Larger prospective trials are needed to establish guidelines for patient selection and risk stratification, particularly concerning ischemic events.

## Source of Support

The authors received no funding for this study.
